# Case Report: Patient-specific 3D-printed preoperative simulation for tailored resection and reconstruction in complex vertebral tumors: a case of giant recurrent chondrosarcoma at the cervicothoracic junction

**DOI:** 10.3389/fsurg.2026.1738809

**Published:** 2026-04-24

**Authors:** M. De Robertis, P. P. Cotrufo, N. Khaled Mansour, P. Oliva, C. Cappelli, E. Stucchi, A. Baram, G. Capo, U. Cariboni, G. Mercante, M. Fornari, F. Pessina, C. Brembilla

**Affiliations:** 1 Department of Biomedical Sciences, Humanitas University, Milan, Italy; 2 Department of Neurosurgery, IRCCS Humanitas Research Hospital, Milan, Italy; 3 IRCCS Humanitas Research Hospital, Milan, Italy; 4 Division of Thoracic Surgery, IRCCS Humanitas Research Hospital, Milan, Italy; 5 Otorhinolaryngology Unit, IRCCS Humanitas Research Hospital, Milan, Italy

**Keywords:** 3D-printed model, anterior column reconstruction, personalized medicine, preoperative planning, vertebral tumors

## Abstract

**Introduction:**

Three-dimensional (3D) printing is a rapidly evolving technology that is transforming various fields and its application in surgery, particularly in spinal procedures, has seen substantial growth in the last 10 years. It enables the production of highly accurate, patient-specific custom implants and anatomical models, enhancing preoperative surgical planning and intraoperative decision-making. This article describes the workflow adopted to produce a 3D-printed model of the cervical column of a patient affected by a recurrent giant cervical chondrosarcoma, focusing on its application in the presurgical resection and reconstruction planning.

**Methods and results:**

We present the case of a 67-year-old female patient with recurrent clear cell chondrosarcoma of the cervical spine. After multidisciplinary discussion, a two-stage (posterior and anterior stage) intentional Enneking inappropriate subtotal resection, followed by adjuvant proton beam therapy (PBT), was planned. One week before the second surgical stage, a surgical simulation was performed on a 3D-printed model. For the 3D virtual modeling, contrast-enhanced CT images of the cervicothoracic spine were obtained for the segmentation of the different anatomical structures. A PolyJet J850 Digital Anatomy® (Stratasys, USA) printer was used due to its ability to assign different materials to each structure, closely mimicking real tissue properties. Surgery was completed without complications, with neurological improvement from American Spinal Injury Association (ASIA) C to D. Adequate decompression and stable reconstruction were achieved. Adjuvant PBT was delivered postoperatively. At the 6-month follow-up, imaging demonstrated good local control and early fusion, and the patient was pain-free and functionally independent.

**Discussion and conclusion:**

The creation of patient-specific, 1:1 scale 3D-printed anatomical models is a crucial tool in the improvement of preoperative planning, providing crucial tactile and visual insights for complex spinal tumor resection and reconstruction, thereby improving surgical precision and safety.

## 1 Introduction

Three-dimensional (3D) printing is a rapidly evolving technology that is transforming various fields, including medicine and surgery, and its application in surgery, particularly in spinal procedures, has seen substantial growth in the last 10 years (1–3). Its integration into healthcare has been driven by progress in manufacturing techniques, cross-sectional imaging, the accessibility of user-friendly medical 3D computer-aided design (CAD) software, and an expanding range of clinical applications (2). Before printing, patient-specific anatomical data are gathered through imaging modalities such as CT, MRI, or X-rays and used to generate a 3D virtual model, which is transferred to a printer and reproduced (1).

3D printing in spine surgery enables the production of highly accurate, patient-specific surgical guides and anatomical models, enhancing preoperative surgical planning and intraoperative decision-making and allowing the development of custom 3D-printed implants (1, 2, 4, 5). Notably, 3D printing has shown significant potential in intricate oncologic resections, such as for malignancies extending along multiple vertebral levels or involving the sacrum, requiring personalized approaches and complex spinal column reconstructions (2, 6).

This article describes the workflow adopted to produce a 3D-printed model of the cervical column of a patient affected by a recurrent cervical chondrosarcoma, treated with an intentional subtotal resection (STR) and reconstructed through a long-segment posterior arthrodesis and a carbon fiber-polyetheretherketone (PEEK) anterior column reconstruction (ACR), focusing on its application in the presurgical resection and reconstruction planning.

## 2 Methods and results

### 2.1 Clinical presentation

**Figure 1:**
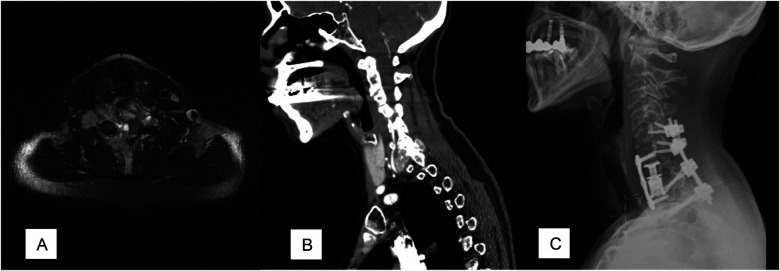
**(A)** Initial presentation of a 67-year-old female patient with recurrent clear cell chondrosarcoma of the cervical spine. MRI shows an enhancing lesion disrupting C7 and invading the spinal canal; **(B)** angio-CT shows the anterior dislocation of the right vertebral artery and the tumoral matrix characterizing the lesion; **(C)** postoperative X-rays. The patient underwent GTR and posterior decompression, followed by circumferential stabilization. This reconstruction comprised an anterior cervical approach using an expandable titanium cage with C6–T1 plating, supplemented by posterior instrumentation spanning C5 to T2.

**Figure 2:**
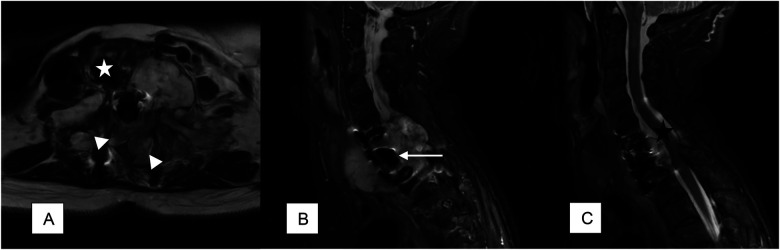
MRI shows massive tumor growth 11 years after the first intervention. **(A)** The lesion extends to the whole C7 residual vertebra, also involving the adjacent C6 and T1, and is characterized by a bilateral high-grade invasion of the spinal canal (white arrowheads) and an anterior exophytic growth on the left side dislocating the trachea (white star) controlaterally; **(B,C)** the tumor entirely envelopes the previously implanted expandable cage (white arrow) and compressed and dislocated the spinal cord (black star).

A 67-year-old female presented to our institution with recurrent clear cell chondrosarcoma of the cervical spine. In 2013, the patient underwent a gross total resection (GTR) and posterior decompression, followed by circumferential stabilization. This reconstruction comprised an anterior cervical approach using an expandable titanium cage with C6-T1 plating, supplemented by posterior instrumentation spanning C5 to T2. Imaging at initial presentation and after the first surgery is presented in Figure 1. Subsequently, from October 2014 to March 2015, she received four cycles of ifosfamide and adriamycin due to a right anterolateral paravertebral recurrence. Radiotherapy (66 Gy in 33 fractions) was administered from November 2015 to January 2016, with the purpose of local control due to a further volumetric increase after the systemic therapy. After these treatments, she reported a motor deficit (MRC 3/5) in her right hand. Good local disease control was achieved until 2023, when the patient complained of gradually worsening mechanical cervical pain (NRS 8/10) radiating to the left scapular region, mild gait ataxia, urinary hesitancy, and a sensation of postvoid residual. In addition, she reported progressive distal paresis in her left upper limb. Electromyography confirmed a newly diagnosed chronic C5–C7 radiculopathy on the left side. At this time, the patient was ambulatory with assistance but not capable of performing any work-related activities [Eastern Cooperative Oncology Group (ECOG) Performance Status 2; American Spinal Injury Association (ASIA) Impairment Scale C]. MRI revealed a significant volumetric increase of the tumor, which presented as a large left anterolateral paravertebral mass causing a grade 2 epidural spinal cord compression (ESCC) (Figure 2). The lesion enveloped the previously implanted titanium hardware and involved the adjacent C6 and T1 vertebral bodies.

### 2.2 Treatment planning and workflow

#### 2.2.1 Surgical strategy

A two-stage (posterior and anterior) intentional Enneking inappropriate (EI) STR, followed by adjuvant PBT, was planned after the tumor board’s multidisciplinary discussion.

**Figure 3:**
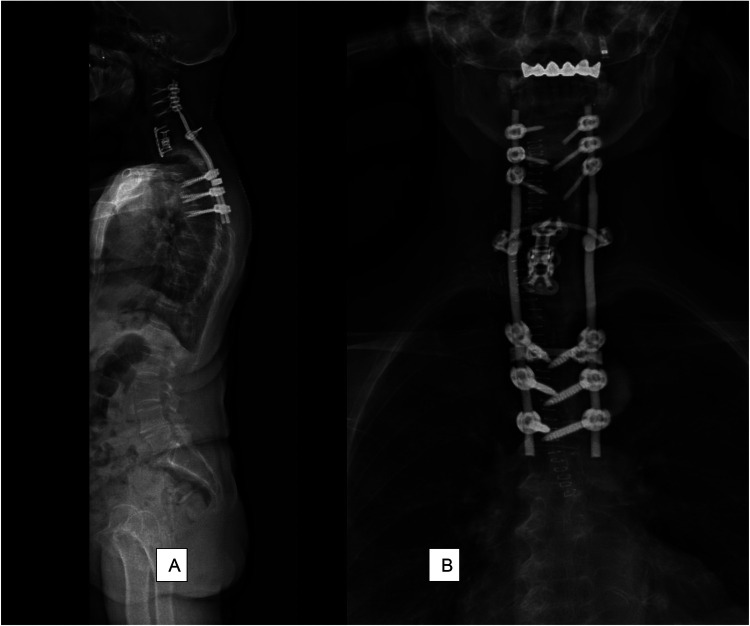
Postoperative standing X-rays. The first surgical stage comprises the extension of the posterior hardware from C4 to T6. Two cervicothoracic rods are used to connect the cervical and thoracic pedicle screws with two cross-links to enhance the strength of the construct. **(A)** Sagittal plane X-ray; and **(B)** coronal plane X-ray.

C6–T1 decompression and separation surgery (SS) were performed in the *first stage*. After removal of the previous posterior hardware, C3–T6 titanium fixation with pedicle screws and rods was achieved; bone fusion was promoted through a fibular omoplastic allograft embedded between the remaining posterior vertebral elements and two titanium cross-links (Figure 3).

The *second anterior stage* was planned 1 month later, during which the patient was referred to an intensive physical therapy program. Good neurological improvement was achieved (from ASIA class C to D).

The planned STR using the anterior approach, combined with ACR, was simulated on a 3D-printed model 1 week before the second surgical stage.

#### 2.2.2 3D virtual model

**Figure 4:**
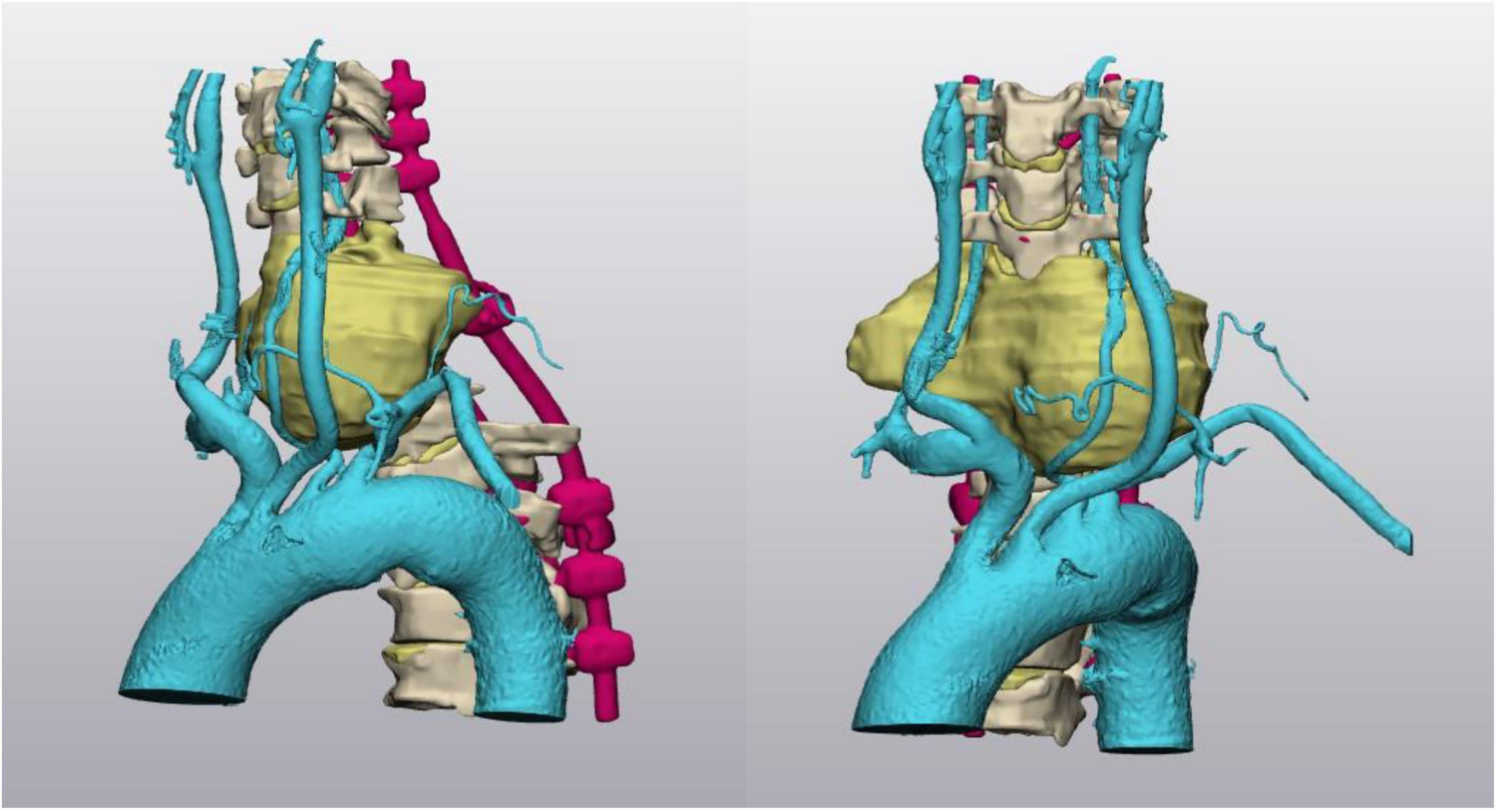
3D virtual model. The tridimensional locoregional anatomy is reconstructed using 3D CAD software. Blue indicates the arteries, yellow indicates the lesion, and magenta indicates the posterior hardware implanted in the previous surgical stage. As depicted in this reconstruction, the left vertebral artery is anteriorly and laterally dislocated by the chondrosarcoma.

**Figure 5:**
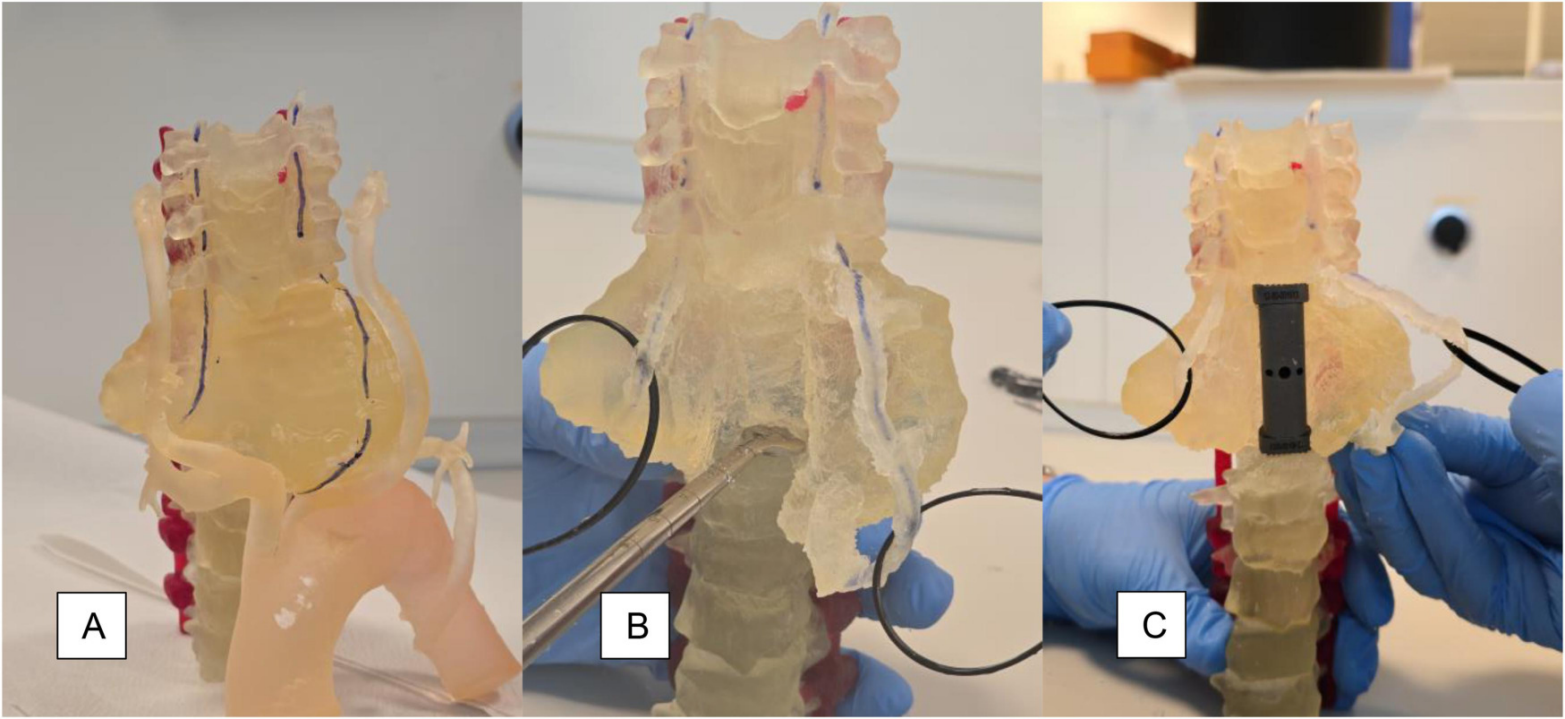
**(A)** A 3D-printed anatomical model is produced; the course of the vertebral arteries on the ventro-lateral surface of the tumor is highlighted in blue; **(B)** the debulking of the lesion and preparation of the somatic plates are simulated; **(C)** after measurement of the residual gap between the C5 inferior plate and the T2 superior plate, we verify the best-fitting cage for the ACR.

Contrast-enhanced CT (CE-CT) images (slice thickness: 0.625 mm; bone and tissue protocol, including angio-CT) of the cervicothoracic spine were obtained for the segmentation of the different anatomical structures. The images were acquired in the supine position, accurately reproducing the operative setting. Manual segmentation was performed on the CT scans to ensure higher model resolution, without merging with MRI images, in order to minimize errors arising from metallic artifacts caused by the posterior hardware implanted during the first surgical stage. Nevertheless, a subsequent qualitative assessment was conducted by comparing CT and MRI datasets. One of the surgeons (PC) and one engineer (NM) contoured the lesion, cervical vasculature, vertebrae, intervertebral discs, and posterior hardware in 3D CAD software, Materialise Mimics (Leuven, Belgium) (Figure 4), to produce an accurate anatomical reproduction. The previously implanted expandable cage was not segmented separately, as it was entirely incorporated into the tumor. A proportional 3D-printed plastic model of the spine from C3 to T6 was produced (Figure 5A). A second surgeon validated the preprinting plan (MR) together with two engineers (PO and CC).

#### 2.2.3 3D printing

A PolyJet J850 Digital Anatomy (Stratasys, USA) printer was used due to its ability to assign different materials to each structure (Figure 4). This PolyJet printer sprays tiny droplets of liquid photopolymer resin onto a build platform and instantly cures them with UV light. Eight materials were loaded into the printer, namely, Agilus30Clear, Agilus30Magenta, BoneMatrix, GelMatrix, TissueMatrix, VeroClear, and VeroPureWhite. The slicing software, GrabCAD, provides a collection of presets that closely mimic the mechanical properties of different anatomical regions. For the model, the following presets were used:

- Bone: Vertebra Clear; this preset uses materials that replicate the hardness of bones, allowing drilling and other invasive procedures.
- Vertebral arteries: Vessel Wall Slightly Compliant Magenta; this preset creates a colored, compliant structure, allowing the possibility to both distinguish and manipulate the vessels.
- Intervertebral discs: Intervertebral Disc Normal; this preset is characterized by softer materials, distinguishable from the bone structure, with a certain level of vertebral segment motion.
- Posterior screws: Vero Magenta; this preset is used for colored rigid material.
- Tumor: Facet Joints Moderately Stiff; this preset was chosen due to its ability to mimic the fibro-cartilaginous texture of the tumor.

The build plate was automatically optimized in terms of material consumption and printing time by tuning the position and direction of the model, reducing the time taken to 1 day and 3 h. The printing process was followed by a postprocessing phase consisting of the manual removal of the support material. Finally, the printed model underwent a washing process in the cleaning station, in a water, caustic soda, and sodium metasilicate solution.

#### 2.2.4 Surgical simulation

One week before the scheduled surgery, a simulation was performed on the 3D-printed model. Tumor debulking and C6–T1 corpectomy were simulated (Figure 5B). The anterior defect was measured to choose the best-fitting implant. A 50-mm-long intersomatic cage produced by Icotec (Icotec-Kong®, Icotec AG, Altstaetten, Switzerland) was selected and verified (Figure 5C). The simulation using the 3D-printed physical model provided tactile insights that were not reproducible through a simple virtual simulation, allowing for the manipulation of materials with textures and consistencies closely resembling *in vivo* tissues. The initial physical assessment of the anatomical relationships between the tumor, the adjacent vertebral bone, and the vascular structures—enabled by a 360° view of the anatomy—enhanced surgical confidence during the actual intraoperative approach, both for the resection and reconstruction.

#### 2.2.5 Surgical technique (second anterior stage)

Selective arterial embolization (SAE) of the ascending cervical branch of the left thyro-cervical trunk was performed prior to the second stage.

**Figure 6:**
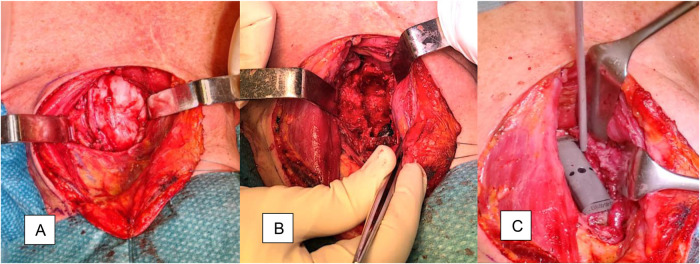
Intraoperative views. **(A)** After dissection of the superficial planes, the tumor capsule is identified; it is possible to identify the left vertebral artery embedded on its surface. **(B)** Piecemeal debulking and removal of the previous hardware allows the identification of the C5–T2 vertebral plates. **(C)** A 50-mm carbon fiber-PEEK cage (Kong, Icotec®) perfectly fits the anterior column defect, as defined during the simulation stage.

For the real surgery, a left paramedian anterior cervical approach, with the assistance of an ear, nose, and throat (ENT) specialist and a thoracic surgeon, was selected. A longitudinal incision was performed along the medial margin of the left sternocleidomastoid. The lesion was identified (Figure 6A) and intralesionally debulked (Figure 6B) in a piecemeal fashion, from C6 to D1. The previous surgical hardware (plate and somatic expandable cage) was removed. The inferior C5 and superior D2 endplates were prepared to enable arthrodesis. A 50 mm carbon fiber-PEEK cage (Kong, Icotec®) and a 67 mm carbon fiber-PEEK anterior cervical plate (ACP, Icotec®) were placed, achieving good alignment (Figure 6C). The surgery was performed under continuous intra-operative neurophysiological monitoring (IONM) and with intraoperative 3D fluoroscopy guidance (O-arm, Medtronic®).

### 2.3 Postoperative course

Patient mobilization was promoted on the first postoperative day. No adverse events were registered. Early progressive improvement in left upper limb sensibility and strength was reported. A postoperative CT scan showed correct positioning of the anterior carbon fiber-PEEK and posterior titanium hardware with the posterior bone graft roof. The patient was discharged 8 days after the second surgical stage.

### 2.4 Adjuvant therapy

Eight weeks after the surgeries, the patient received PBT for the residual lesion. The dose administered was 70 Gy in 35 fractions. After PBT, a mild worsening of the left-hand motor deficit was registered [Common Terminology Criteria for Adverse Events (CTCAE) Version 5.0, Grade 2].

### 2.5 Follow-up and outcome

**Figure 7:**
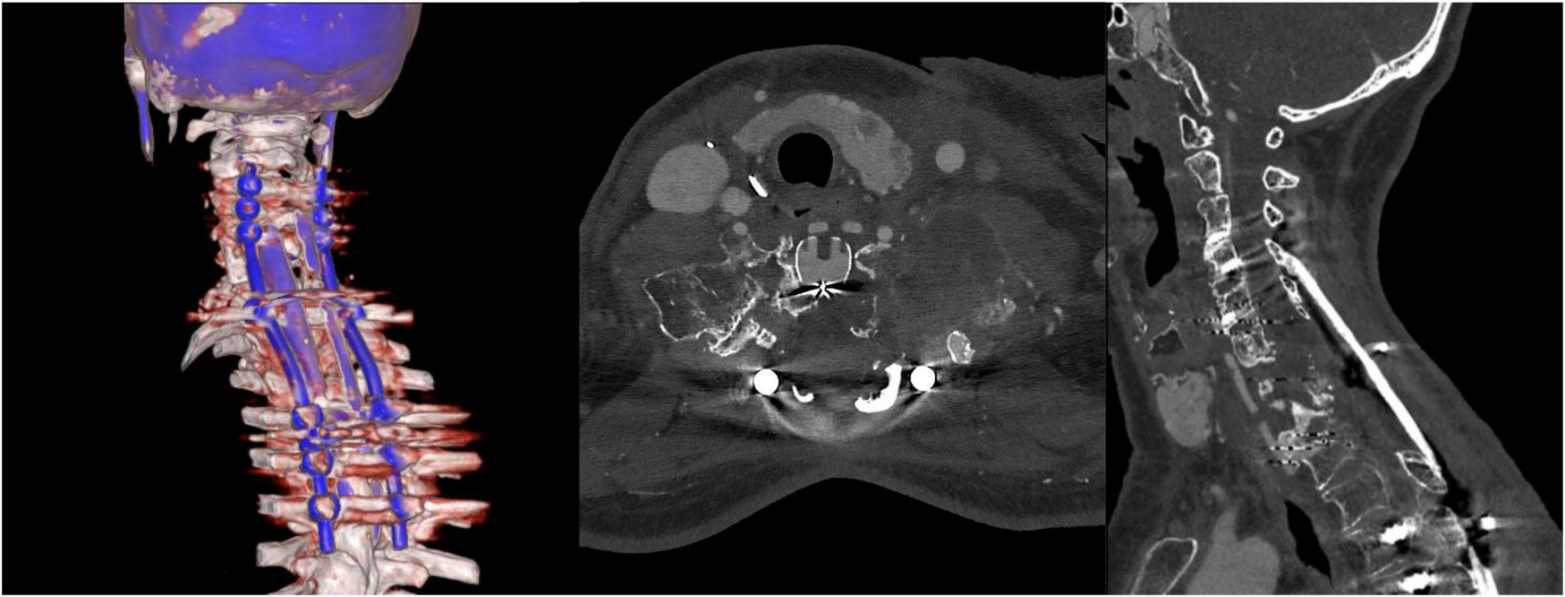
At the 6-month follow-up, a CE photon-counting CT scan shows good local control (with signs of tumor necrosis) and good alignment of the cervicothoracic spine. In the sagittal plane, initial signs of posterior bone fusion are visible between the posterior fibular homoplastic allograft and the vertebral laminae.

The 6-month follow-up CE-CT scan showed good local control and initial signs of posterior bone fusion (Figure 7). The patient, categorized as ASIA D, presented without axial or radicular pain and resumed her daily activities with a good degree of autonomy.

## 3 Discussion

The successful management of this complex case of recurrent cervical chondrosarcoma underscores the pivotal role of 3D printing in modern spinal surgery. The creation of a patient-specific, 1:1 scale anatomical model was instrumental in our preoperative planning, providing unparalleled tactile and visual insight into the distorted anatomy resulting from both the tumor recurrence and previous surgical interventions. This enabled a meticulous strategy for the en bloc resection and the subsequent multilevel reconstruction. As surgical procedures become less invasive, the value of detailed preoperative planning increases significantly, and 3D models provide a crucial advantage in this context (7).

Our experience aligns with a growing body of literature that champions 3D printing as a transformative tool for complex spinal pathologies (3, 8). Initially used for preoperative visualization, its applications have expanded to include surgical education, intraoperative guides, and custom implants (4, 5). The primary advantage, as demonstrated here, is the enhancement of preoperative surgical planning, which is crucial in cases with challenging anatomy, such as pediatric instrumentation, congenital anomalies, or complex tumor resections (9–11). This detailed planning phase can translate into improved intraoperative efficiency, greater surgical accuracy, and enhanced safety (12, 13).

While our case utilized a 3D model for planning, the application of this technology extends further. The use of 3D-printed surgical guides has been shown to significantly increase the accuracy of screw placement, reduce operative time, minimize blood loss, and decrease radiation exposure (13, 14). Moreover, for large vertebral defects following tumor resection, such as in our patient, the next frontier is the use of 3D-printed patient-specific implants (6, 12). These customized prostheses can address complex anatomical voids where conventional implants are inadequate, potentially offering superior stability and biomechanical performance (15). Reports confirm that custom-designed implants can be easily positioned, facilitating the surgical procedure and reducing the need for complex intraoperative graft fashioning (16, 18, 19).

In summary, 3D-printed anatomical models are rapidly evolving from a novelty to an indispensable tool in complex spine surgery. They empower surgical teams to better understand intricate pathologies, refine their operative approach, and anticipate reconstructive challenges (3, 5). As the technology continues to advance, particularly in the realm of biocompatible materials, custom implants, and the integration with biologics, 3D printing promises to further increase the precision, safety, and personalization of treatment for patients with the most challenging spinal conditions (16–19).

## 4 Conclusion

The presented clinical case and the related management workflow emphasize the potential role of 3D-printed anatomical models in the improvement of preoperative planning, providing crucial tactile and visual insights for complex spinal tumor resections and reconstruction, thereby improving surgical precision and safety.

## Data Availability

The original contributions presented in the study are included in the article/Supplementary Material, further inquiries can be directed to the corresponding author.
